# Minimally Invasive Surfactant Therapy: An Analytical Report of Our Prospective Dubai Cohort

**DOI:** 10.7759/cureus.8455

**Published:** 2020-06-05

**Authors:** Karthikeyan Gengaimuthu

**Affiliations:** 1 Neonatology, Medeor Hospital, Dubai, ARE

**Keywords:** surfactant, minimally invasive surfactant therapy, less invasive surfactant administration, preterm, neonate, respiratory distress syndrome, retinopathy of prematurity, sepsis, intraventricular hemorrhage, continuous positive airway pressure

## Abstract

Introduction: Type 2 pneumocytes of the respiratory epithelium secrete the endogenous surfactant, a detergent-like substance that lines the alveolar sacs of the lungs. The surfactant facilitates the gas exchange process across the alveolar membrane by preventing the collapse of the alveoli and thereby maintaining their distended state. Respiratory distress syndrome of the premature neonates is characterized by quantitative and/or qualitative defects of endogenous surfactant metabolic pathways. The advent of exogenous surfactant therapy is rightly hailed as the major milestone in advancement of the care of the babies with surfactant-deficient lung disease. The administration of exogenous surfactant traditionally involves endotracheal intubation and mechanical ventilation. Minimally invasive surfactant therapy (MIST) is the technique of delivering surfactant without intubation whilst continuing the baby on noninvasive respiratory support. This author introduced MIST as the default way of administering surfactant in his neonatal units in Dubai and has to his credit the first published report on MIST from the United Arab Emirates in this journal in 2018.

Objective: To analyze prospectively all our babies in Dubai who received surfactant by MIST.

Design: Prospective descriptive study of all babies receiving surfactant by MIST starting from January 2018.

Setting: Three tertiary care neonatal centers in Dubai.

Patients and methods: Thirteen babies (gestation 27-36 weeks and birth weight 0.95-2.81 kg) were treated with MIST on 15 occasions. Catheterization techniques were by infant feeding tube in 10 babies, LISA (less invasive surfactant administration) catheter in one baby, and 2.0 size endotracheal tube (ETT) with surfactant filled syringe directly attached to its hub two times each in two babies. Curosurf the porcine surfactant at 200 mg/kg was used on nine occasions and Survanta the bovine surfactant at 4 mL/kg on six occasions.

Main outcome measures: MIST success defined as the baby not needing intubation and ventilation within 72 hours post MIST. Outcome measures with respect to the different modalities of MIST procedure and surfactant preparations used in this prospective cohort.

Results: Only one of the 13 babies (7.7%) in this cohort needed escalation of support with mechanical ventilation and high frequency oscillation (HFO). MIST using semi-rigid catheters like the LISA catheter or the smallest size ETT was technically easier to perform. No differences were observed with regard to the surfactant preparation used. None had an abnormal neurosonogram and there were no instances of sepsis and necrotizing enterocolitis either. The baby that had an unsuccessful MIST had retinopathy of prematurity that was effectively treated with Laser post discharge from neonatal unit. All the babies in this cohort had age appropriate developmental milestones on subsequent follow up visits ranging from three months to two years.

Conclusions: MIST can be easily mastered and adapted in our neonatal units. MIST by any of the three variations of techniques as described in our cohort at FiO2 thresholds not exceeding 0.4 results in quicker resolution of the surfactant deficient lung disease, reduces the oxygen days in these babies and perhaps thereby insures intact survival of these babies.

## Introduction

Noninvasive respiratory support is currently the accepted strategy of neonatal care and its basic philosophy is to maximize the alveolar area available for gas exchange by minimizing atelectrauma [1-3]. Inherent to this strategy are the early initiation of continuous positive airway pressure (CPAP) and appropriate and optimum use of surfactant to prevent the need for intubation and prolonged ventilation thus avoiding the additional insults of barotraumas and volutrauma [4-6]. Surfactant needs to reach the distal airways to be effective which meant intubation was needed for its administration with consequent mechanical ventilation for a variable period [6]. INSURE (INtubate, SURfactant , Extubate) strategy was evolved in Scandinavian countries that minimized the duration of ventilation post intubation and surfactant administration to four to six minutes and continuation of baby on noninvasive support and this significantly reduced the need for mechanical ventilation [7-9]. Further evolution of this towards avoiding the airway injury led to the introduction of MIST (minimally invasive surfactant therapy) and this has been widely assimilated in clinical practice by European neonatologists [10-14]. We introduced MIST for surfactant delivery in our neonatal units in Dubai in 2018 and have published our initial experience of the first three cases in 2018 [15] and subsequently our experience with 2.0 endotracheal tube (ETT) as an improvized novel technique for MIST in 2019 [16]. This article presents our perspectives of MIST from the insight we have gained from our cohort of babies that received surfactant by MIST from January 2018.

## Materials and methods

From January 2018, this author introduced MIST as the default technique for surfactant therapy in the neonatal units of Dubai wherein he worked as the clinical lead for neonatal care. The respiratory support guidelines for our neonates include initiation of nasal CPAP at the earliest point of identification of distress, monitoring FiO2, SpO2 and capillary blood gases and FiO2 thresholds of 0.3-0.4 for surfactant therapy. Of the 49 preterm neonates with respiratory distress managed by our team during this period, 13 babies needed surfactant administration (15 times, once each in 11 babies and twice in two babies) and we performed MIST on all these occasions. Informed consent for the procedure and surfactant use was obtained from parents. The MIST procedure on every instance was performed by this author, who being the consultant neonatologist had the privileges for performing this procedure by the regulatory authorities in Dubai, United Arab Emirates.

In this MIST cohort of 13 babies (gestational age 27-36 weeks and birth weight 0.95-2.81 kg) all babies had hyaline membrane disease and one of them had critical congenital heart disease in addition. Catheterization techniques were by infant feeding tube (6F Microtech, USA) in 10 babies, LISA (less invasive surfactant administration) catheter (Chiesi Pharmaceuticals, Italy) in one baby, and by 2.0 mm endotracheal tube with syringe directly attached to its hub two times in two babies. Curosurf 200 mg/kg and Survanta 4 mL/kg (dosages rounded upwards to conform to vials available) were used on nine and six occasions each respectively. Premedication (IV morphine 100 mcg/kg) was used in only one baby in this cohort (35 weeks, 2.115 kg male neonate). We used the Magill forceps to guide the feeding tube on that single occasion only and were able to comfortably pass the infant feeding tube without unassisted by Magill forceps in the other nine babies.

To ensure that the surfactant has indeed been delivered appropriately without significant reflux (>15% of the volume) we used a simple bedside method. We aspirated the stomach before the procedure and during the procedure flushed the surfactant down to disperse by air flush until there was no regurgitation noted in the catheter or the tubes used for the procedure. Post withdrawal of the MIST conduit we checked the gastric aspirate on every occasion to quantify visible surfactant reflux to stomach. During the procedure the baby was monitored using two multichannel monitors and hypoxia was avoided by adjusting the settings of CPAP and FiO2.

Ethical considerations

Informed consent for the procedures was obtained and documented in each instance as mandated by Joint Commission International (JCI) standards. Informed consent was also obtained from the parents by the first author for using patient data in an anonymized manner in medical journals. As this is only a case series report of our neonates receiving surfactant by MIST that is an accepted superior way of surfactant delivery that was incorporated in our clinical practice in full compliance with JCI standards, this report was deemed not to require an Institutional Review Board consideration.

## Results

Table 1 summarizes the demographics, clinical course, and the clinical outcome of these babies. The 13 babies who constitute the Dubai MIST cohort includes the initial three cases we had reported in 2018 on a pilot basis in this journal [14]. The procedure was successfully performed on all the occasions without the requirement for intubation in the traditional way as required for INSURE technique. In all the instances this was completed within eight minutes while the baby was continued on the nasal CPAP support. All the babies in this cohort except a 28 weeks baby had recovered from the respiratory distress without any further escalation of support. The 28 weeks baby in whom MIST failed was intubated and ventilated 36 hours post MIST for persistent pulmonary hypertension of the newborn and had air leaks that were drained. This baby required further escalation of care, an additional dose of Survanta during intubation for ventilation on day 7 and subsequent high frequency oscillation (HFO) and oxygen therapy or more intensive respiratory care for a total of 15 oxygen days.

**Table 1:** The Dubai MIST cohort. MIST, minimally invasive surfactant therapy; LISA, less invasive surfactant therapy; nCPAP, nasal continuous positive airway pressure; ETT, endotracheal tube; ROP, retinopathy of prematurity.

Serial no	Gestation (weeks)	Birth weight (kg)	Sex	Age @ MIST (hours)	Surfactant preparation	Need for escalation of support, end of oxygen treatment, or total duration of oxygen days	Outcome, age at last assessment, ROP status
1.	30+6	1.03	Male	16	Survanta (feeding tube)	No, nCPAP x 28 h post MIST	Intact survival, 2 years, No ROP
2.	35+3	2.115	Male	5	Curosurf (feeding tube)	No, nCPAP X 2 days post MIST	Intact survival, 2 years, No ROP
3.	28+1	1.18	Female	9	Curosurf (feeding tube)	Yes, HFO, PPHN, Pneumothoraces, repeat survanta on day 7 by ETT, total of 15 oxygen days	Survived. 2 years, Laser for ROP III x once
4.	32+1	1.75	Male	6	Survanta (feeding tube)	No, nCPAP x 24 h post MIST	Intact survival, 2 years, No ROP
5.	34	2.4	Male	12.5	Curosurf (feeding tube)	No, nCPAP x 2 days post MIST	Intact survival, 2 years, No ROP
6.	27+5	0.95	Female	14	Curosurf (feeding tube)	No, nCPAP x 3 days post MIST	Intact survival, 2 years, No ROP
7.	33	2.0	Male Twin 2	21	Curosurf (LISA catheter)	No, nCPAP x 13 h post MIST to room air	Intact survival, 2 years, No ROP
8.	33	2.1	Male Twin 1	44 (self pay issues)	Curosurf (feeding tube)	No, nCPAP x 33 h post MIST to room air	Intact survival, 2 years, No ROP
9.	34	2.81	Female	13	Survanta (2 size ETT syringe attached direct removing the adapter)		Intact survival, 18 months, No ROP
10.	34 (2^nd^ dose in same baby)	2.81	Female	22	Survanta 2^nd^ dose (same technique as above)	No, nCPAP continued for 1 day post 2^nd^ MIST, Off O2 3 days post 2^nd^ MIST	
11	36+2	2.37	Female	40 h 45	Curosurf 6 mL (feeding tube)	No, off nCPAP 8 h post MIST, off Oxygen 4 days post MIST	Intact survival, 15 months, no ROP
12	32+2	2.24	Female	12	Curosurf 6 mL (feeding tube)	No, 20 h post MIST weaned off CPAP and O2	Survival, 12 months, no ROP
13	29+3	1.39	Male	4 h	Survanta 8 mL By 2.0 ETT	No diagnosed as having single ventricle morphology CCHD as baby did not improve even after two doses of Survanta by MIST. Referred to cardiac center	Survival, 9 months, no ROP
14	29+3 (same baby as in 13)	Same	Male	10 h	Survanta 8 mL By 2.0 ETT		
15	32+3	1.93	Female	12 h	Curosurf 480 mg	No, 9 h post MIST weaned off CPAP and O2 stopped at 24 h age	Intact survival, 3 months, no ROP

 Procedural ease and the early clinical course until the time of discharge from the neonatal unit were not influenced by the surfactant preparation used. One significant observation was that with adequate air flush volumes post MIST, no reflux during or post MIST was noted even with higher volumes of surfactant that preparations like Survanta require. MIST using semi-rigid catheters like the LISA catheter or the smallest size ETT in this cohort was technically easier to adapt in clinical practice. All babies in our neonatal unit had survived during this period. None in this cohort had an abnormal neurosonogram (intraventricular hemorrhage 3 or above, periventricular leukomalacia, etc.). All the babies had passed their hearing screening tests by the Auditory Brainstem Response technique. Developmental and growth milestones were assessed to be appropriate for the corrected age in each of these babies during the three months to two years follow up visits. 

## Discussion

We are presenting our analysis of neonates who received surfactant by MIST in the Dubai corporate hospitals wherein the author was the consultant and lead for neonatal care. Favorable evidence towards MIST as an inherent constituent of the noninvasive schema of management of respiratory distress in premature infants is accumulating rapidly [16-19]. A recent meta-analysis of a pool of 895 neonates treated with MIST concluded that this technique significantly reduced the composite outcome of death or bronchopulmonary dysplasia (BPD) at 36 weeks, BPD at 36 weeks among survivors, the need for mechanical ventilation within 72 hours of birth as well as the need for mechanical ventilation anytime during the neonatal intensive care unit stay [19]. Out of 13 MIST babies in our cohort only one had MIST failure and needed intubation and further escalation of support as discussed earlier. Reported rate of MIST failure (needing intubation and mechanical ventilation within 72 hours post procedure) is 30% even in the extremely preterm neonates and this is strikingly better than the 69% reported with the INSURE technique [20-21]. In our Dubai cohort the MIST failure rate is 7.7% and hence we believe that this reported 30% MIST failure rate can further be improved by using the higher recommended dose of Curosurf and performing MIST at lower FiO2 thresholds for the more immature babies.

 The baby that had MIST failure in our cohort had retinopathy of prematurity (ROP) that was appropriately managed with Laser treatment thus ensuring that she had clear unaided vision on the 18 months follow up examination. All the babies in our MIST cohort have been assessed to have physical growth and developmental milestones appropriate for their corrected age on the three months to two years follow up visits post discharge.

 A 53% relative risk reduction in the incidence of severe grades of intraventricular hemorrhage (IVH) in the MIST group versus the intubated group was noted in an earlier study [19] and this is further reinforced by a recent study reporting that even within the MIST group those neonates who had MIST failure and were intubated have significantly increased risk for severe IVH (14.3% versus 3.1%, adjusted odds ratio 3.8) [20]. None of our MIST babies had IVH grade 2 or above. There were no instances of culture proven sepsis in our cohort. In the large German Neonatal Network cohort of 7533 preterm neonates with gestation ranging from 22 to 28 weeks, 6319 (83.9%) received surfactant, 41.5% (2624 of 6319) by LISA, and 58.5% (3695 of 6319) by ETT [22]. LISA brings about significant reduction in mortality and also the serious preterm specific morbidities IVH, BPD, sepsis, and ROP [22]. The observed increase in Focal Intestinal Perforation in LISA group babies that is significant for the subgroup less than 26 weeks in this cohort implies that either CPAP by the interfaces used and the pressure settings thereof was non beneficial (higher pressure could have compromised the vulnerable intestinal perfusion) or more rigorous standards of care to avoid CPAP belly coupled perhaps with lower accepted abdominal girth standards is needed in this subgroup.

We have previously in 2018, reported our initial successful experience with three babies that received MIST by infant feeding tube as the conduit [14] and introduced MIST as the default way of administering surfactant hence after. It has to be noted that we performed MIST by infant feeding tube (6 Fr size, Microtech Inc, USA) unaided (without the use of Magill forceps on nine of the 10 the odd being the first baby) receiving MIST this way and given the technical ease with which we performed this no sedation or narcotics were needed. MIST with LISA catheter was technically easier to perform and this avoided even the brief period of desaturation and bradycardia that was observed post MIST procedure in our initial cases. As LISA catheter may not be readily available in all units, we later performed an improvised MIST procedure using the smallest size ETT 2.0, whose inner diameter is equivalent to 8 Fr infant feeding tube and instilled surfactant directly from a syringe attached to the hub of the ETT. Infant feeding tubes are usually made of polyurethane material that is much more flexible and hence difficult to guide into the trachea in contradistinction to the poly vinyl chloride material that is in ETT [23-24]. We have earlier reported that this improvised MIST equivalent of the semirigid catheter technique (the prototype is the LISA catheter) is technically simpler to adapt in clinical practice [15]. This observation is in conformity with earlier reports from Europe [23-24].

Reflux and wastage of surfactant especially the higher dose volume preparations like Survanta is a valid concern with a logical basis [25-26] and it was held that concentrated surfactant preparations like Curosurf will fare better in MIST procedure. We noticed in our MIST cohort that with air flush post MIST as described in this article subjects and methods section was effective in ensuring that surfactant irrespective of volume factor is maximally effective [15]. More recently a report from Canada attests to this feasibility and successful outcome with MIST using high dosage volume surfactant, 5 mL/kg of BLES (bovine lipid extract surfactant) and MIST failure rate was only 15% [27].

It has to be noted that whilst European units are the leaders in the implementation of this minimally or less invasive way of surfactant administration acceptance and adaption of this technology in care practices is lower in other countries like Japan, USA, and the United Kingdom. In a 2019 Web-based survey of 150 neonatal units in England it was reported that only 11% are using LISA for surfactant therapy [28]. Nevertheless 78% of the units were considering implementation of LISA after the bottlenecks of lack of adequate skill levels of the care givers and incorporation in clinical care guidelines are addressed to [28].

## Conclusions

Minimally invasive surfactant therapy has become the default way of surfactant administration in European units and has proven advantages over the INSURE procedure. This author introduced the MIST for surfactant therapy in the United Arab Emirates and incorporated this as the default way for surfactant therapy in the neonatal units wherein he was the clinical lead for neonatal care. MIST using infant feeding tube can be performed without using Magill forceps by post acquisition of the appropriate skill level and experience and the need for sedation or narcotic analgesia minimized to a great extent. MIST by whatever variations of techniques as described in our cohort and at FiO2 thresholds not exceeding 0.4 results in a quicker resolution of the surfactant deficient lung disease, reducing the oxygen days in these babies and perhaps thereby insuring intact survival of these babies.

## References

[1] Sweet DG, Carnielli V, Greisen G (2019). European Consensus Guidelines on the management of respiratory distress syndrome - 2019 update. Neonatology.

[2] American Academy of Pediatrics: Committee on Fetus and Newborn (2014). Respiratory support in preterm neonates at birth. Pediatrics.

[3] Hillman N, Jobe AH (2013). Noninvasive strategies for management of respiratory problems in neonates. Neoreviews.

[4] Schmölzer GM, Kumar M, Pichler G, Aziz K, O'Reilly M, Cheung PY (2013). Non-invasive versus invasive respiratory support in preterm infants at birth: systematic review and meta-analysis. BMJ.

[5] Guay JM, Carvi D, Raines DA, Luce WA (2018). Care of the neonate on nasal CPAP: a bedside guide. Neonatal Netw.

[6] Sardesai S, Biniwale M, Wertheimer F, Garingo A, Ramanathan R (2017). Evolution of surfactant therapy for respiratory distress syndrome: past, present, and future. Pediatr Res.

[7] Bohlin K, Gudmundsdottir T, Katz-Salamon Katz-Salamon, M M, Jonsson B, Blennow M (2007). Implementation of surfactant treatment during continuous positive airway pressure. J Perinatol.

[8] Blennow M, Bohlin K (2015). Surfactant and noninvasive ventilation. Neonatology.

[9] Ramanathan R (2007). Early surfactant therapy and noninvasive ventilation. J Perinatol.

[10] Herting E, Härtel C, Göpel W. (2020). Less invasive surfactant administration (LISA): best practices and unanswered questions. Curr Opin Pediatrics.

[11] Gyu-Hong S (2017). Update of minimally invasive surfactant therapy. Korean J Pediatr.

[12] Barkkuff WD, Soll RF (2019). Novel surfactant administration techniques: will they change outcome?. Neonatology.

[13] Klotz D, Porcaro U, Fleck T, Fuchs H (2017). European perspective on less invasive surfactant administration—a survey. Eur J Pediatr.

[14] Gengaimuthu K (2018). Should minimally invasive surfactant therapy be a must in neonatal intensive care units? Pilot report of initial cases in Dubai. Cureus.

[15] Gengaimuthu K (2019). Minimally invasive surfactant therapy using a 2.0 mm uncuffed endotracheal tube as the conduit: an easily adaptable technique. Cureus.

[16] Lau CSM, Chamberlain RS, Sun S (2017). Less invasive surfactant administration reduces the need for mechanical ventilation in preterm infants: a meta-analysis. Global Pediatric Health.

[17] Aldana-Aguirre JC, Pinto M, Featherstone RM, Kumar M (2017). Less invasive surfactant administration versus intubation for surfactant delivery in preterm infants with respiratory distress syndrome: a systematic review and meta-analysis. Arch Dis Child Fetal Neonatal Ed.

[18] Isayama T, Iwami H, McDonald S, Beyene J (2016). Association of noninvasive ventilation strategies with mortality and bronchopulmonary dysplasia among preterm infants: a systematic review and meta-analysis. JAMA.

[19] Kribs A, Roll C, Göpel W (2015). Non intubated surfactant application vs conventional therapy in extremely preterm infants: a randomized clinical trial. JAMA Pediatr.

[20] Ce Janssen L, Van Der Spil J, van Kaam AH, Dieleman JP, Andriessen P, Onland W, Niemarkt HJ (2019). Minimally invasive surfactant therapy failure: risk factors and outcome. Arch Dis Childhood Fetal Neonatal Ed.

[21] Brix N, Sellmer A, Jensen MS, Pedersen LV, Henriksen TB (2014). Predictors for an unsuccessful INtubation-SURfactant-Extubation procedure: a cohort study. BMC Pediatr.

[22] Härtel C, Paul P, Hanke K (2018). Less invasive surfactant administration and complications of preterm birth. Sci Rep.

[23] Rigo V, Debauche C, Maton P, Broux I, Van Laere D (2017). Rigid catheters reduced duration of less invasive surfactant therapy procedures in manikins. Acta Paediatrica.

[24] Fabbri L, Klebermass-Schrehof K, Aguar M (2018). Five-country Manikin Study found that neonatologists preferred using the LISAcath rather than the angiocath for less invasive surfactant administration. Acta Paediatrica.

[25] De Luca D, Minucci A, Gentile L, Lapoluongo ED (2014). Surfactant inadvertent loss using feeding catheter or endotracheal tube. Am J Perinatol.

[26] Fujioka K, Kuroda J, Yamone K, Lijme K, Morioka I (2017). Loss of surfaten@ during bolus administration using a feeding catheter. Pediatrics Int.

[27] Bhattacharya S, Read B, McGovern E, da Silva O (2019). High-volume surfactant administration using a minimally invasive technique: Experience from a Canadian neonatal intensive care unit. Paediatr Child Health.

[28] Bhayat S, Kaur A, Premadeva I, Reynolds P, Gowda H (2020). Survey of less invasive surfactant administration in England, slow adoption and variable practice. Acta Paediatrica.

